# Chemical and Bioinformatics Analyses of the Anti-Leishmanial and Anti-Oxidant Activities of Hemp Essential Oil

**DOI:** 10.3390/biom11020272

**Published:** 2021-02-12

**Authors:** Luigi Menghini, Claudio Ferrante, Simone Carradori, Marianna D’Antonio, Giustino Orlando, Francesco Cairone, Stefania Cesa, Antonello Filippi, Caterina Fraschetti, Gokhan Zengin, Gunes Ak, Massimo Tacchini, Kashif Iqbal

**Affiliations:** 1 Department of Pharmacy, Università degli Studi “Gabriele d’Annunzio”, Via dei Vestini 31, 66100 Chieti, Italy; luigi.menghini@unich.it (L.M.); simone.carradori@unich.it (S.C.); giustino.orlando@unich.it (G.O.); 2 Bioinvest S.r.l., Via Filippo Masci, Building 6, 66100 Chieti, Italy; dantoniomarianna2@gmail.com; 3 Department of Drug Chemistry and Technology, Sapienza University of Rome, 00185 Rome, Italy; francesco.cairone@uniroma1.it (F.C.); stefania.cesa@uniroma1.it (S.C.); antonello.filippi@uniroma1.it (A.F.); caterina.fraschetti@uniroma1.it (C.F.); 4 Department of Biology, Science Faculty, Selcuk University, Campus, 42130 Konya, Turkey; gokhanzengin@selcuk.edu.tr (G.Z.); akguneselcuk@gmail.com (G.A.); 5 Department of Life Sciences and Biotechnology (SVeB), UR7 Terra&Acqua Tech, University of Ferrara, 44121 Ferrara, Italy; 6 Department of Pharmacy, University of Lahore, Islamabad Campus, Islamabad 54590, Pakistan; kashifiqbal321@gmail.com

**Keywords:** *Cannabis sativa* L., essential oil, GC-MS analysis, scavenging/reducing activity, *Leishmania tropica*, in vivo studies, bioinformatics

## Abstract

Industrial hemp is a multiuse crop that has been widely cultivated to produce fibers and nutrients. The capability of the essential oil (EO) from inflorescences as antimicrobial agent has been reported. However, literature data are still lacking about the hemp EO antiprotozoal efficacy in vivo. The present study aims to unravel this concern through the evaluation of the efficacy of hemp EOs (2.5 mL/kg, intraperitoneally) of three different cultivars, namely *Futura 75*, *Carmagnola selezionata* and *Eletta campana*, in mice intraperitoneally infected with *Leishmania tropica*. A detailed description of EO composition and targets-components analysis is reported. Myrcene, α-pinene and *E*-caryophyllene were the main components of the EOs, as indicated by the gas-chromatographic analysis. However, a prominent position in the scenario of the theoretical interactions underlying the bio-pharmacological activity was also occupied by selina-3,7(11)-diene, which displayed affinities in the micromolar range (5.4–28.9) towards proliferator-activated receptor α, cannabinoid CB2 receptor and acetylcholinesterase. The content of this compound was higher in *Futura 75* and *Eletta campana*, in accordance with their higher scavenging/reducing properties and efficacy against the tissue wound, induced by *L. tropica*. Overall, the present study recommends hemp female inflorescences, as sources of biomolecules with potential pharmacological applications, especially towards infective diseases.

## 1. Introduction

Industrial hemp is a multiuse crop that has been widely cultivated for the production of fibers and nutrients. Moreover, there is a great deal of interest about the extraction of hemp secondary metabolites, including terpenes, terpenophenolics and flavonoids, which could be at the basis of its health-promoting effects [1]. The Italian Government promoted industrial hemp cultivation as a strategy to implement environment-friendly crops and to contrast the loss of agricultural lands, a phenomenon that is particularly evident in central Italy, due to an unbalance between industrial- and agricultural-regulating policies. In the Abruzzo region (central Italy), hemp cultivation is the object of renewed interest, which is witnessed by the increased surface dedicated to hemp crops. For these purposes, a huge number of hemp varieties have been selected in the last two decades, characterized by low ∆^9^-tetrahydrocannabinol (THC) content (<0.2%). Hemp cultivation is mainly focused to the production of fibers and seeds, whereas the aerial parts, floral buds and leaves, are considered as waste or by-products [1,2]. During the last years, however, inflorescences are becoming innovative and high-quality by-products of the whole hemp chain, employed for preparations of tonic beverages (tea infusion) or as flavorings and nutraceuticals in certified food and supplements, respectively [3]. This is also suggested by recent in vitro studies highlighting the capability of both hemp water extract and essential oil (EO) as promising antioxidant, anti-inflammatory, anti-proliferative and antimicrobial agents [4,5,6,7,8,9]. Anyway, the antimicrobial spectrum appeared to be wider for the EO, particularly against pathogenic bacterial strains. The composition and antimicrobial activity of the EO of three hemp cultivars, namely *Carmagnola selezionata*, *Fibranova* and *Futura 75*, were investigated [7] and the last variety showed the highest EO yield and antimicrobial activity. This could be related, at least partially, to the presence of terpene compounds, such as α-pinene, myrcene, trans-β-ocimene, γ-terpinolene, *E*-caryophyllene and α-humulene. The EO of Futura 75 was also described as a promising insecticide against larvae of *Spodoptera littoralis* and *Culex quinquefasciatus* as well as against adults of *Musca domestica* [2], whereas the good tolerability demonstrated in a pilot clinical study [10], further suggested the use of industrial hemp EOs as a novel insecticide with low ecological impact on the environment [11]. Despite there being several studies highlighting hemp antimicrobial activity in vitro, literature data are still lacking about the hemp antiprotozoal efficacy in in vivo paradigm. Therefore, the present study aims to unravel this concern through the evaluation of the efficacy of hemp EO of three different cultivars, *Futura 75*, *Carmagnola selezionata* and *Eletta campana*, in adult male BALB/c mice intraperitoneally infected with *Leishmania tropica* [12]. The present cultivars were selected according to their consolidated crop production, in the Abruzzo region. The qualitative phytochemical fingerprint, including color analysis, terpene fraction, scavenging/reducing and metal-chelating properties, was investigated, as well. Additionally, a bioinformatics analysis, including network pharmacology and virtual screening, was conducted to predict the mechanism of action underlying the observed bio-pharmacological effects.

## 2. Materials and Methods

### 2.1. Chemicals for Antileishmanial Activity

Amphotericin-B, antibiotics (streptomycin and penicillin), dimethyl sulfoxide, fetal bovine serum, growth media (RPMI-1640) were purchased from Merck (Darmstadt, Germany). All the materials used in experimental process was of analytical grade.

### 2.2. Experimental Farm and Plant Extraction

Three selected cultivars of industrial hemp, namely *Cannabis sativa* L. cultivar ‘*Futura 75*’ (F75), *C. sativa* L. cultivar ‘*Eletta campana*’ (EC) and *C. sativa* L. cultivar ‘*Carmagnola selezionata*’ (CS) were cultivated in experimental crops, in the Abruzzo region and more specifically in the surrounding of the Pescara river valley, Italy (Table 1). This valley is a typical piedmont-hilly zone of the central-eastern Apennines, which originates from the ecotone between the Gran Sasso and Maiella-Morrone massifs, in the northern and southern part, respectively and declines within 45 km in an extended drainage basin to the Adriatic coast. The basin area expresses the Mediterranean bi-climate characters typical of the Adriatic coast with dry summer characterized by law rainfall. The total extension of the experimental crops were 24,000 square meters on level ground, with a mean slope lower than 5% and altitude range 70–100 m asl. The planting scheme was in lines with a central 2 m irrigation strip widths.

In order to improve the development of plants according to the high temperature and long daylight, the sowings were performed between mid and late March 2019, with a prevision to reach a scalar blooming stage, ready for harvesting, in late August-September. The cultivar EC is a dioecious Italian selection deriving from original crops of the early 1900s, largely cultivated in the south of Italy for fiber production. It is in the European community list of varieties admitted for cultivation and its agronomic needs fit with the Mediterranean environment. It is considered as a late flowering cultivar that produces high amount of biomass, due to the large leaf surface and compact smelling inflorescences, endowed with a rich presence of terpenes. The cultivar name refers to the Campania region and the selected cultivar is largely cultivated throughout central and South Italy. Similarly, CS is a dioecious cultivar originally selected for the production of fiber. The mature plants can grow over 6 m but are also adapted for seeds and flowers production. *Futura 75* is a French selection of monoecious variety for seeds and biomass production, characterized by high adaptability for multi-purpose cultivation. Previous experiences of cultivation confirmed the suitability of this cultivar for local environmental conditions, also in central Italy [9]. All selected seeds are present in the plant variety database of the European Commission in the agricultural species list. The genus *Cannabis* and specifically the selected cultivars are characterized by high adaptability and, based on previous experiences, no specific agronomical practices were planned but just a superficial soil preparation (ploughing, disc harrowing and milling), automated sowing for optimized seed density, no fertilization and only three programmed automated irrigation cycles. During the phase of flowering, an accurate manual collection of only mature flowers was done. The collection was repeated daily to obtain only high-quality mature flowers. Fresh plant material was immediately transferred to the laboratory and extracted within 6 h. Random samples from collections were selected and botanical identity was confirmed by classical taxonomical approach (done by Prof. L. Menghini, Full Professor in Pharmaceutical Botany at the Department of Pharmacy, “G. d’Annunzio” University, Chieti, Italy). Fresh flowers were distributed to obtain a homogeneous dispersion in the distillation chamber in a stainless-steel distillatory Clevenger-type apparatus (Albrigi Luigi S.r.l., Stallavena, Italy). The volumetric EO yields (%, *v*/*w*) were determined and expressed for phytochemical (mL in plant fresh weight) or agronomical (mL from hectare) interpretations. Samples of EOs to be used for analytical characterization and biological tests were dehydrated through a passage in anhydrous sodium sulfate and stored in a dark glass bottles with polyethylene terephthalate (PTFE) septa cap at 4 °C until used.

### 2.3. Colorimetric Determination of Scavenging/Reducing and Metal-Chelating Properties

Scavenging/reducing effects were investigated by radical scavenging 2,2-diphenyl-1-picrylhydrazyl (DPPH) and 2,2′-azino-bis(3-ethylbenzothiazoline-6-sulfonic acid) (ABTS) and reducing power (CUPRAC and FRAP) assays and results were expressed as trolox equivalents (TE). The metal-chelating properties were expressed as ethylenediaminetetraacetic (EDTA) equivalents. Details of the assays were reported in previous studies of ours [4,8].

### 2.4. Color Analyses

The hemp essential oil samples were submitted to colorimetric analyses and monitored for color parameters (L*, a*, b*, C*_ab_ and *h*_ab_) by means of a colorimeter X-Rite SP-62 (X-Rite Europe GmbH, Regensdorf, Switzerland), equipped with an integration sphere. The cylindrical coordinates C*_ab_ and *h*_ab_ were calculated. Results were expressed as the mean value ± SD [13,14].

### 2.5. Gas Chromatography/Mass Spectrometry (GC/MS) Analysis

The volatile organic compounds (VOCs) analysis was carried out using an Agilent Technologies 6850 gas chromatograph (Santa Clara, CA, USA) coupled with an Agilent Technologies 5975 mass spectrometer (Santa Clara, CA, USA), equipped with an HP-5MS capillary column (5% phenyl 95% methylpolysiloxane, 30 m × 0.25 mm inner diameter, film thickness 0.25 µm; Hewlett-Packard, Palo Alto, CA, USA). About 1 μL of the filtered oil has been manually injected in the split configuration mode and GC parameters were adjusted as follows: injector temperature, 250 °C; flow rate of the helium carrier gas (99.995% purity), 1.0 mL/min. The oven temperature was set at 40 °C (5 min), then raised to 200 °C at 5 °C/min and maintained at this temperature for 60 min. MS parameters were set as follows: energy of electron ionization, 70 eV; solvent delay, 6 min; source temperature, 230 °C quadrupole temperature, 150 °C; and mass scan was carried out over the 50–350 *m*/*z* range. The eluted compounds were identified by matching the relative mass spectra with those available from both a commercial database (FFNSC 3, Chromaleont srl, Messina, Italy) and online libraries (NIST 11, Flavor2, Scientific Instrument Services, Ringoes, NJ, USA). Kovats index (KI) was used as a second parameter to confirm the analyte identification. KIs were measured using a mixture of n-alkanes (C8–C24) in the same analytic conditions and then compared with values reported in the literature and in the FFNSC 3 database. The identity of several compounds was confirmed through injection of standard samples available from commercial sources. The relative abundance of oil components was obtained by integrating the GC/MS peak areas without any further correction as previously reported [15].

### 2.6. Preparation of Culture for Leishmania Parasites

Promastigotes of *L. tropica* (KWH23) were cultured in RPMI-1640 medium along with 10% fetal bovine serum, penicillin (200 U/mL) and streptomycin (0.2 mg/mL). The details about the employed protocol are reported in previous study of ours [12].

### 2.7. Preparation of Sample Solution

To prepare sample solution, aliquots (12.5 µg) of EC, CS and F75 EOs were mixed in 1 mL saline solution containing 0.5% *v*/*v* dimethyl sulfoxide (DMSO) [16]. The use and percentage of DMSO as co-solvent were according to literature data [17]. The same authors reported that intraperitoneal administration of DMSO is well-tolerated by mice up to 10 mL/kg/day dose.

### 2.8. In Vivo Study

The animal model was based on male adult BALB/c mice, supplied by National Institute of Health, Islamabad, Pakistan, whereas Animal and Ethics Committee of the University of Lahore, Islamabad approved current study design for experimental purposes. Standard diet was given to BALB/c mice during experimental plan. For sample administration, BALB/c mice were allocated in 3 groups, the 1st group composed of sample control (2.5 mL/kg, corresponding to 12.5 µg/mL EO in a final volume of 100 µL), the 2nd group had standard drug (positive control: amphotericin B 12.5 mg/kg) and the 3rd group composed of negative control (saline + 0.5% DMSO). Treatments were injected once a day intraperitoneally for 8 weeks. Experimental plan during in vivo study was followed, as previously described [12]. Regarding the EO dose (2.5 mL/kg), the corresponding concentration (12.5 µg/mL in 100 µL) was considered not toxic, based on our previous observations of null effect on multiple cell lines treated with comparable EO concentrations [9] and according to a study using similar DMSO final concentrations in a cell-based model [18]. The DMSO amount administered intraperitoneally to mice is 0.5%, which is comparable to the doses used in papers dealing with other essential oils administered in murine models of leishmaniasis [19,20]. Additionally, multiple studies showed the intraperitoneal administration of terpene compounds, such as *E-*caryophyllene and linalool, at dosages much higher (100–200 mg/kg), than those injected in the present paradigm [21,22].

### 2.9. Bioinformatics and In Silico Analysis

Putative targets were predicted using bioinformatics platforms SwissTargetPrediction and STITCH, whereas the components-targets plot was built using the software Cytoscape version 3.8. Docking calculations were conducted through the Autodock Vina of PyRx 0.8 software, as recently described [23]. Crystal structures of target proteins were derived from the Protein Data Bank (PDB) with PDB IDs as follows: 1GQR [acetylcholinesterase (AChE)], 2P54 [peroxisome proliferator activated receptor α (PPAR-α)], 2HFF [cannabinoid type 2 receptor (CB2)]. Discovery studio 2020 visualizer was employed to investigate the protein-ligand non-bonding interactions.

### 2.10. Statistical Analysis

The statistical significance of difference between controls and experimental groups was evaluated through analysis of variance (ANOVA), followed by Dunnett’s post hoc test. Results were expressed as means ± SD (standard deviation). Statistical analysis was performed using GraphPad Prism™ software (Version 5.01; GraphPad Software, San Diego, CA, USA). A *p* value < 0.05 was considered as statistically significant.

## 3. Results and Discussion

Many factors influence the productive performances of hemp plants in terms of essential oil yield and quality, including environmental conditions, climatic factors and agronomical practices. Furthermore, the selection of raw plant material could be critical, particularly when the distillation is applied as a strategy to valorize byproducts. The secretory glands are mainly concentrated in floral structures and technical manipulation or mechanical processes can significantly affect quantitative and qualitative yield. In this study, the manual collection of mature inflorescences allows obtaining a high-quality plant material with yields in essential oils ranging among 0.85 and 1.33 EO/kg fresh flowers for EC and CS, respectively. The order of magnitude agrees with previous published data, also if a direct comparison is not easy to perform, due to the different cultivars, variability of operative conditions, different plant parts used, distillation of fresh/dry rather than raw/shredded material, harvesting periods and others [24,25,26]. The performances of experimental cultivation are summarized in Table 2, whereas the Table 3 summarizes the intrinsic scavenger/reducing properties of the three EOs. Specifically, *Futura 75* and *Eletta campana* EOs showed the highest antiradical activity, in most of the employed colorimetry assays.

**Figure 1:**
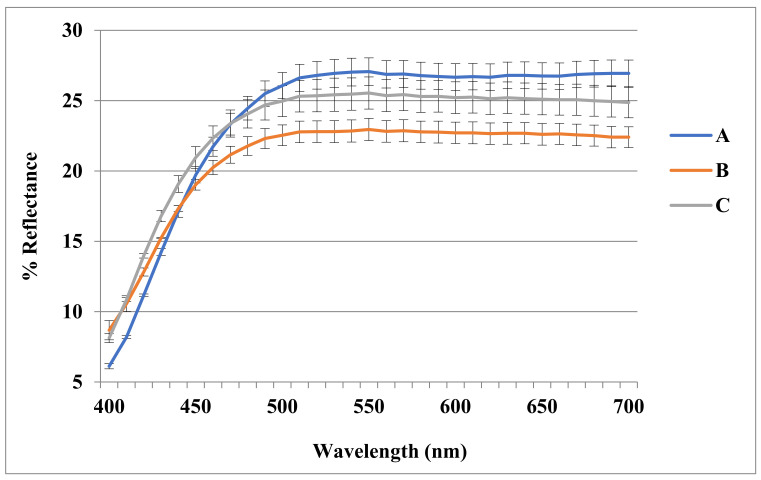
Reflectance curves of hemp essential oils. (A) EC essential oil; (B) CS essential oil; (C) F75 essential oil.

To our knowledge the tristimulus colorimetry, employed in this study to evaluate the color properties of hemp essential oils, was not yet reported in the literature, with the only exception of our previous work [9], in which one essential oil from *Futura 75* and its relative aromatic water were submitted to this evaluation. The three hemp essential oils, slightly yellow colored, were submitted to colorimetric analyses, whose results are shown in Table 4 as well the relative reflectance curves reported in Figure 1. Substantially, samples showed a high L* value, index of their brightness, a less significant yellow parameter b* (among 7.82 and 12.82) and confirmed a weak green coloration denoted by the just negative a* value. Therefore, a significant difference could be shown for the paler yellow nuance. The experimental data are in accordance with already published colorimetric analysis of *Futura 75* essential oil [9], with only relevant change in yellow (b* parameter) that varies from 24.36 in the sample obtained from 2016 to 8.47 in the 2019 sample and highlights the effects of plant variability related to environmental and intrinsic factors.

The comparison between the EOs composition points to a basically uniform pool of the phytochemicals. However, the relative abundance of the detected metabolites significantly diverges throughout the analyzed cultivars (Table 5). More in detail, the metabolite rankings are: myrcene> E-caryophyllene> α-pinene in *Carmagnola selezionata*, E-caryophyllene> α-pinene> myrcene in *Futura 75* and heptacosane > *E*-caryophyllene> α-pinene> myrcene in *Eletta campana*. Myrcene is the main metabolite present in *Carmagnola selezionata* (26.4%), that can be further discriminated by the absence of tetracosane (peak 38), conversely detected in relevant amount in other samples. On the other side, heptacosane (peak 39) is only present in the *Eletta campana* sample (23.9%). The latter contains more complex mixture characterized by the exclusive presence of other six compounds (peaks 16, 17, 25, 26, 30, 33). The quantitative differences as for myrcene (peak 4, range 6.8–26.4%), terpinolene (peak 14, 2.3–7.0%), limonene (peak 9, 1.8–4.7%) and selina-3,7(11)-diene (peak 32, 0.5–2.5%) suggest potentially relevant effects on organoleptic properties. In our previous paper [9], we have investigated the composition of essential oil obtained from *Futura 75* cultivated in the same area. It is evident a quantitative variability of the essential oil within the same variety expressed by the lowest concentration detected for *E*-caryophyllene, caryophyllene-oxide and α-humulene (28%, 15%, and 13%, respectively in our previous paper) [9]. The qualitative variability of this cultivar is evidenced also by comparison with the study of Marini and colleagues [27] that identified α-pinene> myrcene> *E*-caryophyllene (19%, 17%, and 15%, respectively) as main components of EO obtained from central Italy crop. This is also consistent, albeit partially, with the results from the study of Iseppi and colleagues (2019) [28]: myrcene> *E*-caryophyllene> α-pinene (19%, 14%, 8%, respectively). Similarly, limited quantitative variations in main compounds (myrcene, *E*-caryophyllene and α-pinene) were recorded in *Carmagnola selezionata* EOs [10,28]. Other information for each cultivar EO is reported in the supporting information (Tables S1–S3).

**Figure 2:**
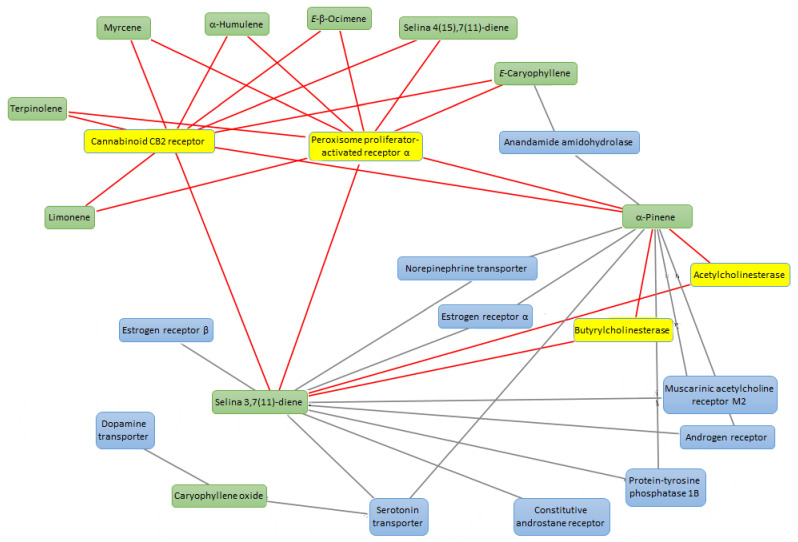
Network pharmacology analysis underlying the theoretical interactions between essential oil phytochemicals, namely α-pinene, α-humulene, *E*-caryophyllene, *E*-β-ocimene, myrcene, terpinolene, limonene, selina-4(15),7(11)-diene, selina-3,7(11)-diene, caryophyllene oxide and multiple mouse target proteins, including acetylcholinesterase, cannabinoid CB2 receptor, peroxisome proliferator-activated receptor α. The molecular targets were identified through the bioinformatics platform SwissTargetPrediction, as well as the components-targets plot was built through Cytoscape software (3.8 version).

**Figure 3:**
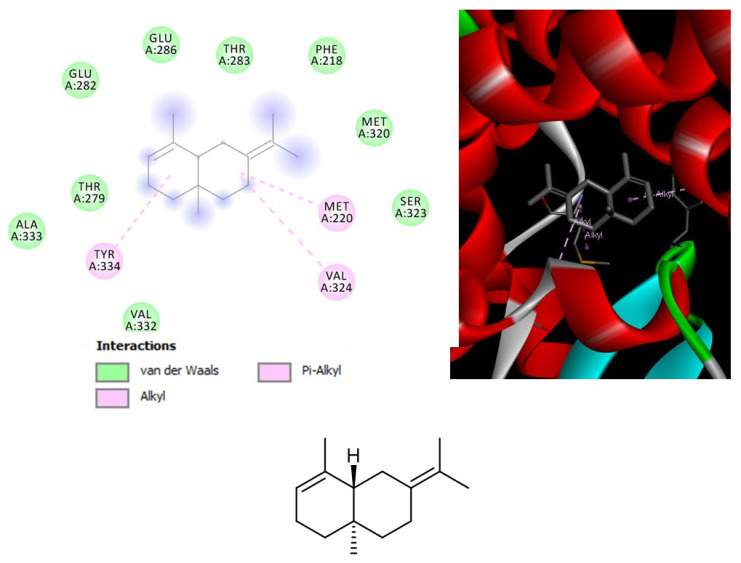
Structure and in silico interactions between selina-3,7(11)-diene and peroxisome proliferator-activated receptor α (PPAR-α; PDB: 2P54). Free energy of binding (ΔG) and affinity (Ki) are −7.2 kcal/mol and 5.4 µM, respectively.

**Figure 4:**
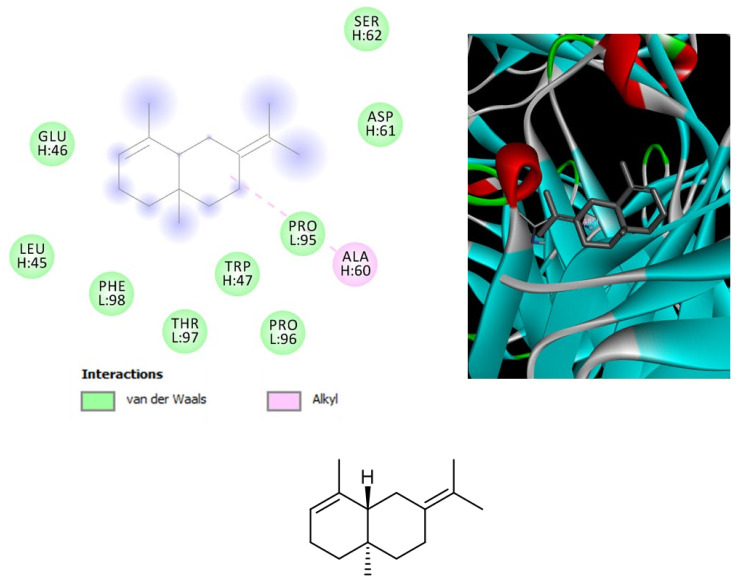
Structure and in silico interactions between selina-3,7(11)-diene and cannabinoid CB2 receptor (CB2; PDB: 2HFF). Free energy of binding (ΔG) and affinity (Ki) are −6.2 kcal/mol and 28.9 µM, respectively.

**Figure 5:**
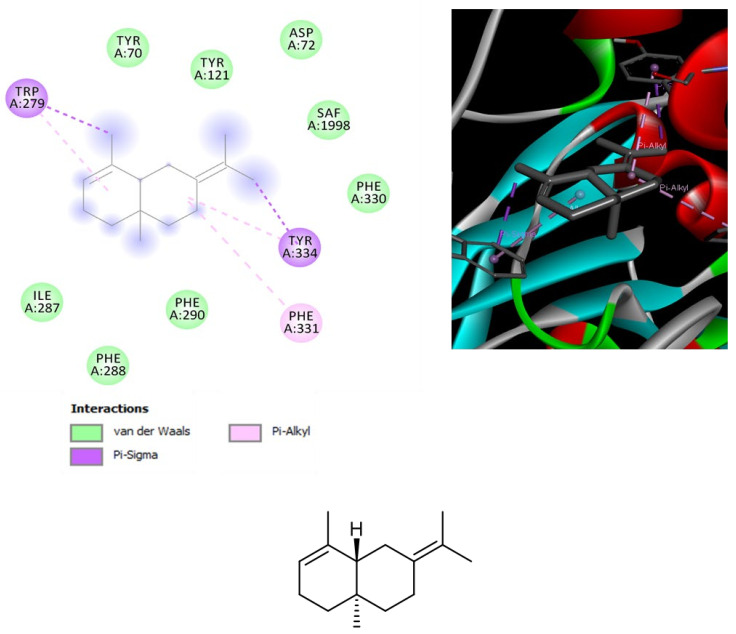
Structure and in silico interactions between selina-3,7(11)-diene and acetylcholinesterase (AChE; PDB: 1GQR). Free energy of binding (ΔG) and affinity (Ki) are −7.2 kcal/mol and 5.4 µM, respectively.

**Figure 6:**
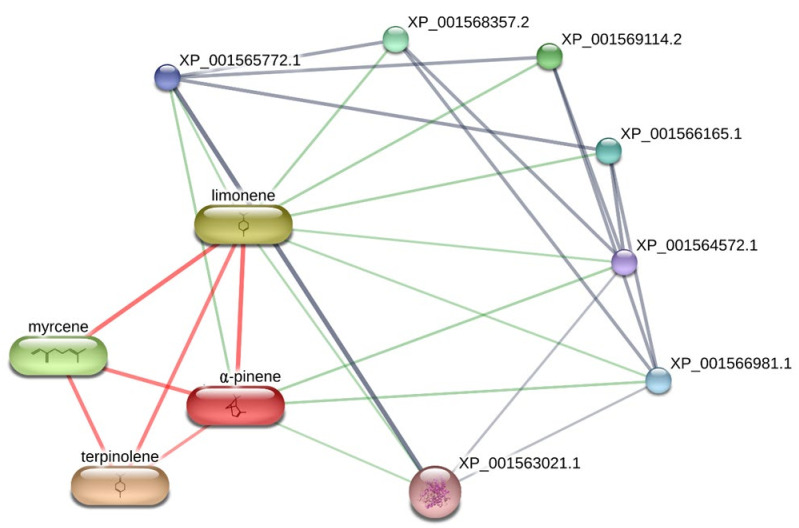
Network pharmacology analysis underlying the theoretical interactions between essential oil phytochemicals, namely α-pinene, myrcene, terpinolene, limonene and leishmanial target proteins. Specifically, the bioinformatics platform showed the prominent position of lanosterol-14α-demethylase (XP_001563021.1), in the scenario of the predicted interactions, namely with α-pinene and limonene. With the only exclusion of XP_001569114.2 (methionine synthase reductase), the other predicted proteins are cytochrome p450-like proteins.

*Leishmania* is a pathogenic parasite (belong to family Trypanosomatidae) which is responsible for worldwide spread of leishmaniasis. As per World Health Organization (WHO) reports, leishmaniasis effected 350 million people of 98 countries in which 12 million people at high risk. Total of 30 strains of *Leishmania* are discovered, 20 of which are lethal. *L. tropica* is one of the identified species which is major cause of cutaneous leishmaniasis in Africa, Asia and Europe. Pentavalent antimonial is the first line therapy against cutaneous leishmaniasis (as per WHO) but due to severe side effects and high cost of drugs, there is need of New lead compounds for treatment purposes [29]. The hemp varieties were also investigated for evaluating their capability in blunting the experimental tissue lesions induced by *L. tropica*, intraperitoneally injected in mice, an in vivo experimental model of visceral leishmaniasis [30]. Among the symptoms of visceral leishmaniosis, there are eye lesions, particularly in those forms induced by *L. tropica* [31]. Therefore, in the present study the efficacy of the hemp essential oils was monitored in terms of reduced eye lesions at the end of the treatment. As depicted in Table 6, after eight weeks of treatment, all cultivars were effective in reducing the dimension of tissue wound, compared to the negative untreated control group (NC). The efficacy of *Eletta campana* and *Futura 75* was similar to that of amphotericin B, that was chosen as the elective and comparative anti-leishmanial treatment [32]. According to the phytochemical analysis, the higher efficacy of *Eletta campana* and *Futura 75* varieties agrees with their best scavenging/reducing profile, with respect to *Carmagnola selezionata*. This is also consistent, albeit partially, with our previous study showing the higher efficacy of *Futura 75* extract as antioxidant and anti-inflammatory agent, compared to *Carmagnola selezionata* [5]. Considering the results of GC-MS, a bioinformatics analysis was conducted on the prominent terpene compounds (>1% total terpene fraction) present in the EOs, as well. The analysis was conducted via SwissTargetPrediction platform and indicated the PPAR-α and CB2 receptors as prominent targets, in the components-targets plot (Figure 2). The activation of the aforementioned enzyme/receptor could mediate, at least in part, the anti-inflammatory properties of terpene compounds [33,34]. The same receptors were also reputed to play a key role in the anti-inflammatory properties induced by cannabidiol, the prominent terpenophenolic compound of industrial hemp [35,36]. Among the identified terpene compounds, selina-3,7(11)-diene was predicted to interact with the aforementioned enzyme/receptor, with putative affinities in the micromolar range (Figure 3, Figure 4 and Figure 5). Moreover, it is sensitive to note that the content of selina-3,7(11)-diene was under the 1%, in the *Carmagnola selezionata* cultivar. Therefore, this could influence the slight lower efficacy of *Carmagnola selezionata* EO, as protective agent in albino mice challenged with *L. tropica*. The bioinformatics analysis conducted via SwissTargetPrediction also indicated the prominent position of AChE, that is a classical target of anti-Alzheimer therapy [37]. Intriguingly, literature data reported simultaneous anti-cholinesterase and anti-leishmanial effects induced by herbal extracts and natural compounds [38,39]. It is well-known that AChE catalyzes the hydrolysis of acetylcholine in choline and acetate. Choline is also the precursor of phosphatidylcholine, a key component of leishmanial membrane [40]. In this context, the inhibition of AChE has been hypothesized as a novel mechanism of anti-leishmanial activity [38]. However, a comparative study including as positive controls both anti-cholinesterase and anti-leishmanial drugs, such as galantamine and miltefosine, respectively, is still missing. Nevertheless, the putative interactions between AChE and selina-3,7(11)-diene, that were predicted to occur at micromolar terpene concentration by the employed virtual screening software (Figure 5), further strengthens the importance to conduct future research in this field. Finally, the in silico predictions conducted via STITCH platform indicated putative interactions between leishmanial lanosterol-14-α-demethylase and different EO terpenes, namely limonene and α-pinene (Figure 6). However, the virtual screening conducted for unravelling the affinity of α-pinene towards lanosterol 14α-demethylase (PDB: 3L4D) rules out a direct effect induced by hemp-deriving terpenes on leishmanial metabolism (putative affinity: Ki > 100 µM).

## 4. Conclusions

The present findings highlight the potential of essential oils from industrial hemp inflorescences as protective agents, in an in vivo model of tissue wound induced by *L. tropica*, in mice. The chemical analyses provide the fingerprint of these selected cultivars and aim at correlating the biological effects with the metabolites identified. The in silico study also evaluates AChE, CB2 and PPAR-α as putative targets mediating the observed protective effects, in mice. Collectively, these data confirm the possibility to use alternative natural compounds in addition to the standard therapy. Considering also recent literature data concerning innovative delivery systems in the anti-leishmanial/anti-oxidant treatment [41,42], we hypothesize the inclusion of the present EOs in nanostructured formulations for improving the efficacy, limiting the administered dosage and thus potentially eliciting the biocompatibility of the lipophilic components of the EOs [43].

## Figures and Tables

**Table 1:** Characteristics of the experimental fields.

Hemp Cultivar	GPS Coordinates	Extension (sqm)	Previous Crops (2 Years)	Sowing Scheme (cm Inter/Intra Lines)	Sowing Density
F75	42,363,059, 14,093,390	4000	Wheat/alfalfa	20 cm/50 cm	15 kg/ha
CS	42,359,222, 14,108,528	14,000	Wheat/Wheat	40 cm/50 cm	20 kg/ha
EC	42,343,824, 14,102,808	6000	Not cultivated	20 cm/40 cm	20 kg/ha

**Table 2:** Agronomical performances of the tested hemp cultivars.

Cultivar	Harvesting Period	Crop Yields		
		Inflorescences (kg Fresh Flowers/ha)	Essential Oil (L/ha)	Hydrodistillation (mL EO/kg Fresh Flowers)
EC (*Eletta campana*)	Late August to early September	1160	0.92	0.85
CS (*Carmagnola selezionata*)	Mid September to mid October	1070	1.39	1.33
F75 (*Futura 75*)	Early to mid September	1000	1.01	0.95

**Table 3:** Scavenging/reducing and metal-chelating properties of the tested hemp essential oils.

EO Cultivar	DPPH (mg TE/g EO)	ABTS (mg TE/g EO)	CUPRAC (mg TE/g EO)	FRAP (mg TE/g EO)	Metal Chelating (mg EDTAE/g EO)	Phosphomolybdenum (mmol TE/g EO)
EC	2.53 ± 0.23 ^a^ *	32.44 ± 0.03 ^a^	45.51 ± 0.75 ^a^	19.29 ± 0.34 ^a^	11.55 ± 0.84 ^a^	17.95 ± 0.34 ^b^
CS	1.18 ± 0.09 ^c^	32.15 ± 0.08 ^b^	29.91 ± 0.95 ^c^	13.55 ± 0.46 ^c^	7.19 ± 0.42 ^b^	17.52 ± 1.12 ^b^
F75	2.11 ± 0.15 ^b^	32.47 ± 0.04 ^a^	35.05 ± 0.85 ^b^	16.16 ± 0.47 ^b^	10.84 ± 0.46 ^a^	18.80 ± 0.47 ^a^

* Values are reported as mean ± SD of three parallel experiments. TE: Trolox equivalent; EDTAE: EDTA equivalent. Different superscripts indicate significant differences in the samples (*p* < 0.05).

**Table 4:** CIEL* a* b* parameters of the hemp essential oils.

	EC	CS	F75
L*	58.39 ± 0.89	54.62 ± 0.79	57.14 ± 1.08
a*	−5.01 ± 0.20	−3.39 ± 0.28	−3.67 ± 0.21
b*	12.82 ± 0.45	7.82 ± 0.70	8.47 ± 0.50
C*_ab_	13.77 ± 0.50	8.52 ± 0.75	9.23 ± 0.55
***h_ab_***	111.34 ± 0.09	113.42 ± 0.16	113.43 ± 0.05

Data are reported as mean value ± SD.

**Table 5:** Gas Chromatography/Mass Spectrometry (GC/MS) analysis of the hemp EOs.

Peak #	Compounds	Area (%)		
		*Eletta campana*	*Futura 75*	*Carmagnola selezionata*
1	α-pinene	11.9	14.9	12.6
2	camphene	0.3	0.3	0.3
3	β-pinene	3.6	3.8	4.1
4	myrcene	6.8	11.8	26.4
5	α-phellandrene	0.1	0.2	0.3
6	∆^3^-Carene	0.5	0.5	0.3
7	α-terpinene	0.1	0.2	0.3
8	*para*-cymene	0.2	0.1	0.2
9	limonene	2.2	1.8	4.7
10	eucalyptol	0.4	0.2	0.5
11	*Z*-β-ocimene	0.5	-	0.4
12	*E*-β-ocimene	1.9	2.9	2.5
13	γ-terpinene	0.2	0.2	0.3
14	terpinolene	2.3	5.1	7.0
15	linalyl anthranilate	0.2	-	0.3
16	fenchyl alcohol	0.1	-	-
17	terpinen-4-ol	0.2	-	-
18	α-ylangene	0.2	-	0.2
19	*Z*-caryophyllene	0.3	0.5	0.4
20	α-*cis*-bergamotene	-	0.3	-
21	*E*-caryophyllene	13.5	19.3	19.1
22	α-*trans*-bergamotene	0.6	1.9	0.2
23	α-humulene	5.3	8.3	7.2
24	9-*epi*-*E*-caryophyllene	0.7	1.1	0.6
25	γ-muurolene	0.5	-	-
26	α-amorphene	0.2	-	-
27	β-selinene	1.5	1.7	1.3
28	α-selinene	1.7	1.3	1.1
29	*Z*-γ-bisabolene	1.4	-	1.1
30	γ-cadinene	0.4	-	-
31	selina-4(15),7(11)-diene	1.3	0.9	0.3
32	selina-3,7(11)-diene	2.5	1.5	0.5
33	germacrene-B	0.3	-	-
34	caryophyllene oxide	2.2	4.3	3.2
35	humulene epoxide	0.7	1.1	1.0
36	α-bisabolol	0.5	-	-
37	*allo*-aromadendrene epoxide	-	0.4	-
38	tetracosane	6.0	8.8	-
39	heptacosane	23.9	-	-
	unknown compounds (n)	4.8 (12)	6.6 (13)	3.6 (10)

n: number of unknown compounds. Compounds are listed in order of increasing KI values (see Tables S1–S3).

**Table 6:** Protective effects induced by the hemp essential oils on tissue wound induced by *L. tropica* challenging, in vivo. Data are expressed as means ± SD.

Sample	Dosing Regimen ^a^	Mean Lesion (mm) before Treatment	Mean Lesion (mm) after Treatment (After 8 Weeks)	% Cure Rate (with 95% Confidence Intervals)	N° of Mice Cured/N° of Mice Infected	Mean Survival Time (Days)
*Eletta campana*	2.5 mL/kg	0.81 ± 0.20	0.31 ± 0.10 ***	96.012 (96.126–98.087)	6/6	≥60
*Futura 75*	2.5 mL/kg	0.73 ± 0.30	0.42 ± 0.70 ***	84.28 (83.25–85.88)	5/6	≥60
*Carmagnola selezionata*	2.5 mL/kg	0.71 ± 0.20	0.61 ± 0.20 **	75.12 (74.86–76.49)	4/6	≥60
Amp	12.5 mg/kg	0.96 ± 0.60	0.34 ± 0.60 ***	95.00 (94.583–96.02)	6/6	≥60
NC ^c^	2.5 mL/kg	0.78 ± 0.50	1.6 ± 0.50	0.000	0/6	≥30

^a^ Route of administration = Intraperitoneally (i.p), ^c^ NC = Negative Control, Amp = Amphotericin B. ANOVA, *p* < 0.001; post hoc test, *** *p* < 0.001, ** *p* < 0.01 vs. NC group.

## Data Availability

The data presented in this study are available on request from the corresponding author.

## Supplementary Materials

The following are available online at: https://www.mdpi.com/2218-273X/11/2/272/s1. Table S1: GC-MS analysis of *Eletta campana* essential oil; Table S2: GC-MS analysis of *Futura 75* essential oil; Table S3: GC-MS analysis of *Carmagnola selezionata* essential oil.

## Sample Availability

Samples of the compounds are available from the authors.

## References

[1] Amaducci S., Scordia D., Liu F.H., Zhang Q., Guo H., Testa G., Cosentino S.L. (2015). Key cultivation techniques for hemp in Europe and China. Ind. Crop. Prod..

[2] Benelli G., Pavela R., Lupidi G., Nabissi M., Petrelli R., Ngahang Kamte S.L., Cappellacci L., Fiorini D., Sut S., Dall’Acqua S. (2018). The crop-residue of fiber hemp cv. Futura 75: From a waste product to a source of botanical insecticides. Environ. Sci. Pollut. Res. Int..

[3] Fernández-Ruiz J., Sagredo O., Pazos M.R., García C., Pertwee R., Mechoulam R., Martínez-Orgado J. (2013). Cannabidiol for neurodegenerative disorders: Important new clinical applications for this phytocannabinoid?. Br. J. Clin. Pharmacol..

[4] Di Giacomo V., Recinella L., Chiavaroli A., Orlando G., Cataldi A., Rapino M., Di Valerio V., Politi M., Antolini M.D., Acquaviva A. (2021). Metabolomic Profile and Antioxidant/Anti-Inflammatory Effects of Industrial Hemp Water Extract in Fibroblasts, Keratinocytes and Isolated Mouse Skin Specimens. Antioxidants.

[5] Ferrante C., Recinella L., Ronci M., Menghini L., Brunetti L., Chiavaroli A., Leone S., Di Iorio L., Carradori S., Tirillini B. (2019). Multiple pharmacognostic characterization on hemp commercial cultivars: Focus on inflorescence water extract activity. Food Chem. Toxicol..

[6] Ingallina C., Sobolev A.P., Circi S., Spano M., Fraschetti C., Filippi A., Di Sotto A., Di Giacomo S., Mazzoccanti G., Gasparrini F. (2020). *Cannabis sativa* L. Inflorescences from Monoecious Cultivars Grown in Central Italy: An Untargeted Chemical Characterization from Early Flowering to Ripening. Molecules.

[7] Nissen L., Zatta A., Stefanini I., Grandi S., Sgorbati B., Biavati B., Monti A. (2010). Characterization and antimicrobial activity of essential oils of industrial hemp varieties (*Cannabis sativa* L.). Fitoterapia.

[8] Orlando G., Recinella L., Chiavaroli A., Brunetti L., Leone S., Carradori S., Di Simone S., Ciferri M.C., Zengin G., Ak G. (2020). Water Extract from Inflorescences of Industrial Hemp Futura 75 Variety as a Source of Anti-Inflammatory, Anti-Proliferative and Antimycotic Agents: Results from In Silico, In Vitro and Ex Vivo Studies. Antioxidants.

[9] Zengin G., Menghini L., Di Sotto A., Mancinelli R., Sisto F., Carradori S., Cesa S., Fraschetti C., Filippi A., Angiolella L. (2018). Chromatographic Analyses, In Vitro Biological Activities and Cytotoxicity of *Cannabis sativa* L. Essential Oil: A Multidisciplinary Study. Molecules.

[10] Gulluni N., Re T., Loiacono I., Lanzo G., Gori L., Macchi C., Epifani F., Bragazzi N., Firenzuoli F. (2018). Cannabis essential oil: A preliminary study for the evaluation of the brain effects. Evid. Based Complement. Alternat. Med..

[11] Regnault-Roger C., Vincent C., Arnason J.T. (2012). Essential oils in insect control: Low-risk products in a high-stakes world. Annu. Rev. Entomol..

[12] Iqbal K., Jamal Q., Iqbal J., Afreen M.S. (2017). Luteolin as a potent anti-leishmanial agent against intracellular *Leishmania tropica* parasites. Trop. J. Pharm. Res..

[13] Cairone F., Carradori S., Locatelli M., Casadei M.A., Cesa S. (2020). Reflectance colorimetry: A mirror for food quality—A mini review. Eur. Food Res. Technol..

[14] Spano M., Di Matteo G., Rapa M., Ciano S., Ingallina C., Cesa S., Menghini L., Carradori S., Giusti A.M., Di Sotto A. (2020). Commercial Hemp Seed Oils: A Multimethodological Characterization. Appl. Sci..

[15] Ingallina C., Capitani D., Mannina L., Carradori S., Locatelli M., Di Sotto A., Di Giacomo S., Toniolo C., Pasqua G., Valletta A. (2020). Phytochemical and biological characterization of Italian “sedano bianco di Sperlonga” Protected Geographical Indication celery ecotype: A multimethodological approach. Food Chem..

[16] Khan M., Ali M., Shah W., Shah A., Yasinzai M.M. (2019). Curcumin-loaded self-emulsifying drug delivery system (cu-SEDDS): A promising approach for the control of primary pathogen and secondary bacterial infections in cutaneous leishmaniasis. Appl. Microbiol. Biotechnol..

[17] Gad S.C., Cassidy C.D., Aubert N., Spainhour B., Robbe H. (2006). Nonclinical vehicle use in studies by multiple routes in multiple species. Int. J. Toxicol..

[18] Smeriglio A., Trombetta D., Alloisio S., Cornara L., Denaro M., Garbati P., Grassi G., Circosta C. (2020). Promising in vitro antioxidant, anti-acetylcholinesterase and neuroactive effects of essential oil from two non-psychotropic *Cannabis sativa* L. biotypes. Phytother. Res..

[19] Monzote L., García M., Scull R., Cuellar A., Setzer W.N. (2014). Antileishmanial activity of the essential oil from *Bixa orellana*. Phytother. Res..

[20] Islamuddin M., Chouhan G., Tyagi M., Abdin M.Z., Sahal D., Afrin F. (2014). Leishmanicidal activities of *Artemisia annua* leaf essential oil against Visceral Leishmaniasis. Front. Microbiol..

[21] Cho H.I., Hong J.M., Choi J.W., Choi H.S., Hwan Kwak J., Lee D.U., Kook Lee S., Lee S.M. (2015). β-Caryophyllene alleviates D-galactosamine and lipopolysaccharide-induced hepatic injury through suppression of the TLR4 and RAGE signaling pathways. Eur. J. Pharmacol..

[22] Caputo L., Reguilon M.D., Mińarro J., De Feo V., Rodriguez-Arias M. (2018). *Lavandula angustifolia* Essential Oil and Linalool Counteract Social Aversion Induced by Social Defeat. Molecules.

[23] Recinella L., Chiavaroli A., di Giacomo V., Antolini M.D., Acquaviva A., Leone S., Brunetti L., Menghini L., Ak G., Zengin G. (2021). Anti-Inflammatory and Neuromodulatory Effects Induced by *Tanacetum parthenium* Water Extract: Results from In Silico, In Vitro and Ex Vivo Studies. Molecules.

[24] Baldini M., Ferfuia C., Piani B., Sepulcri A., Dorigo G., Zuliani F., Danuso F., Cattivello C. (2018). The Performance and Potentiality of Monoecious Hemp (*Cannabis sativa* L.) Cultivars as a Multipurpose Crop. Agronomy.

[25] Bertoli S., Tozzi L., Pistelli L., Angelini L.G. (2010). Fibre hemp inflorescences: From crop-residues to essential oil production. Ind. Crop. Prod..

[26] Fischedick J.T., Hazekamp A., Erkelens T., Choi Y.H., Verpoorte R. (2010). Metabolic fingerprinting of *Cannabis sativa* L., cannabinoids and terpenoids for chemotaxonomic and drug standardization purposes. Phytochemistry.

[27] Marini E., Magi G., Ferretti G., Bacchetti T., Giuliani A., Pugnaloni A., Rippo M.R., Facinelli B. (2018). Attenuation of Listeria monocytogenes Virulence by *Cannabis sativa* L. Essential Oil. Front. Cell Infect. Microbiol..

[28] Iseppi R., Brighenti V., Licata M., Lambertini A., Sabia C., Messi P., Pellati F., Benvenuti S. (2019). Chemical Characterization and Evaluation of the Antibacterial Activity of Essential Oils from Fibre-Type *Cannabis sativa* L. (Hemp). Molecules.

[29] Iqbal K., Iqbal J., Staerk D., Kongstad K.T. (2017). Characterization of Antileishmanial Compounds from *Lawsonia inermis* L. Leaves Using Semi-High Resolution Antileishmanial Profiling Combined with HPLC-HRMS-SPE-NMR. Front. Pharmacol..

[30] Katakura K. (2016). An experimental challenge model of visceral leishmaniasis by *Leishmania donovani* promastigotes in mice. Parasitol. Int..

[31] Mittal A., Ray Y., Soneja M., Chatterjee M., Kumar P. (2020). Concurrent ocular and cutaneous leishmaniasis caused by *Leishmania tropica*. QJM.

[32] Rosenthal E., Delaunay P., Jeandel P.Y., Haas H., Pomares-Estran C., Marty P. (2009). Le traitement de la leishmaniose viscérale en Europe en 2009. Place de l’amphotéricine B liposomale. Med. Mal. Infect..

[33] Gonçalves E.C.D., Assis P.M., Junqueira L.A., Cola M., Santos A.R.S., Raposo N.R.B., Dutra R.C. (2020). Citral Inhibits the Inflammatory Response and Hyperalgesia in Mice: The Role of TLR4, TLR2/Dectin-1 and CB2 Cannabinoid Receptor/ATP-Sensitive K^+^ Channel Pathways. J. Nat. Prod..

[34] Irrera N., D’Ascola A., Pallio G., Bitto A., Mazzon E., Mannino F., Squadrito V., Arcoraci V., Minutoli L., Campo G.M. (2019). β-Caryophyllene Mitigates Collagen Antibody Induced Arthritis (CAIA) in Mice Through a Cross-Talk between CB2 and PPAR-γ Receptors. Biomolecules.

[35] Atalay S., Jarocka-Karpowicz I., Skrzydlewska E. (2020). Antioxidative and Anti-Inflammatory Properties of Cannabidiol. Antioxidants.

[36] Pollastro F., Minassi A., Fresu L.G. (2018). Cannabis Phenolics and their Bioactivities. Curr. Med. Chem..

[37] Moss D.E. (2020). Improving Anti-Neurodegenerative Benefits of Acetylcholinesterase Inhibitors in Alzheimer’s Disease: Are Irreversible Inhibitors the Future?. Int. J. Mol. Sci..

[38] Mogana R., Adhikari A., Debnath S., Hazra S., Hazra B., Teng-Jin K., Wiart C. (2014). The antiacetylcholinesterase and antileishmanial activities of *Canarium patentinervium* Miq. Biomed. Res. Int..

[39] Lenta B.N., Vonthron-Sénécheau C., Weniger B., Devkota K.P., Ngoupayo J., Kaiser M., Naz Q., Choudhary M.I., Tsamo E., Sewald N. (2007). Leishmanicidal and Cholinesterase Inhibiting Activities of Phenolic Compounds from *Allanblackia monticola* and *Symphonia globulifera*. Molecules.

[40] Wassef M.K., Fioretti T.B., Dwyer D.M. (1985). Lipid analyses of isolated surface membranes of *Leishmania donovani* promastigotes. Lipids.

[41] Ray L., Karthik R., Srivastava V., Singh S.P., Pant A.B., Goyal N., Gupta K.C. (2021). Efficient antileishmanial activity of amphotericin B and piperine entrapped in enteric coated guar gum nanoparticles. Drug Deliv. Transl. Res..

[42] Rinaldi F., Hanieh P.N., Longhi C., Carradori S., Secci D., Zengin G., Ammendolia M.G., Mattia E., Del Favero E., Marianecci C. (2017). Neem oil nanoemulsions: Characterisation and antioxidant activity. J. Enzyme Inhib. Med. Chem..

[43] Kumari P., Luqman S., Meena A. (2019). Application of the combinatorial approaches of medicinal and aromatic plants with nanotechnology and its impacts on healthcare. Daru.

