# Supplementary material for: Chemical and Bioinformatics Analyses of the Anti-Leishmanial and Anti-Oxidant Activities of Hemp Essential Oil
Source: Biomolecules. 2021 Feb 12;11(2):272. doi: 10.3390/biom11020272 (PMC7917915; doi:10.3390/biom11020272)
Supplement: Supplementary file 1 [file biomolecules-11-00272-s001.pdf]

# Chemical and bioinformatics analyses of the anti-leishmanial and anti-oxidant activities of hemp essential oil

**Luigi Menghini[1], Claudio Ferrante[1*], Simone Carradori[1], Marianna D'Antonio[2], Giustino Orlando[1], Francesco Cairone[3], Stefania Cesa[3], Antonello Filippi[3], Caterina Fraschetti[3], Gokhan Zengin[4], Gunes Ak[4], Massimo Tacchini[5*], Kashif Iqbal[6]**

[1] Department of Pharmacy, Università degli Studi "Gabriele d'Annunzio", via dei Vestini 31, 66100 Chieti, Italy; luigi.menghini@unich.it (L.M.); claudio.ferrante@unich.it (C.F.); simone.carradori@unich.it (S.C.); giustino.orlando@unich.it (G.O.)
[2] Bioinvest S.r.l., via Filippo Masci, Building 6, 66100 Chieti, Italy; dantoniomarianna2@gmail.com (M.D.A.)
[3] Department of Drug Chemistry and Technology, Sapienza University of Rome, 00185 Rome, Italy; stefania.cesa@uniroma1.it (S.Ce.); francesco.cairone@uniroma1.it (F.C.); caterina.fraschetti@uniroma1.it (C.Fr.); antonello.filippi@uniroma1.it (A.F.)
[4] Department of Biology, Science Faculty, Selcuk University, Campus, 42130 Konya, Turkey; gokhanzengin@selcuk.edu.tr (G.Z.); akguneselcuk@gmail.com (G.A.)
[5] Department of Life Sciences and Biotechnology (SVeB), UR7 Terra&Acqua Tech, University of Ferrara, Ferrara 44121, Italy; massimo.tacchini@unife.it (M.T.)
[6] Department of Pharmacy, The University of Lahore-Islamabad campus, 54590 Islamabad, Pakistan. kashifiqbal321@gmail.com (K.I.)

*Correspondence: Prof. Claudio Ferrante; e-mail: claudio.ferrante@unich.it; Tel.: +39-0871-355-4753; Fax.: Tel. +39-0871-355-4753; Address: Department of Pharmacy, Università degli Studi "Gabriele d'Annunzio", via dei Vestini 31, 66100 Chieti, Italy. Prof. Massimo Tacchini; e-mail: massimo.tacchini@unife.it; Address: Department of Life Sciences and Biotechnology (SVeB), UR7 Terra&Acqua Tech, University of Ferrara, Ferrara 44121, Italy.

**Table S1.** GC-MS analysis of *Eletta campana* essential oil.

| **Compound** | **Area %** | **RI[a]** | **RIL[b]** |
|---|---|---|---|
| α-pinene | 11.9 | 934 | 933 |
| camphene | 0.3 | 949 | 953 |
| β-pinene | 3.6 | 977 | 978 |
| myrcene | 6.8 | 994 | 991 |
| α-phellandrene | 0.1 | 1006 | 1007 |
| $\Delta^3$-carene | 0.5 | 1011 | 1009 |
| α-terpinene | 0.1 | 1019 | 1018 |
| *para*-cymene | 0.2 | 1028 | 1024 |
| limonene | 2.2 | 1031 | 1030 |
| eucalyptol | 0.4 | 1040 | 1032 |
| *Z*-β-ocimene | 0.5 | 1043 | 1035 |
| *E*-β-ocimene | 1.9 | 1053 | 1046 |
| γ-terpinene | 0.2 | 1062 | 1058 |
| terpinolene | 2.3 | 1091 | 1086 |
| linalyl anthranilate | 0.2 | 1110 | 1104 |
| fenchyl alcohol | 0.1 | 1123 | 1123 |
| terpinen-4-ol | 0.2 | 1187 | 1184 |
| α-ylangene | 0.2 | 1378 | 1371 |
| *Z*-caryophyllene | 0.3 | 1414 | 1413 |
| *E*-caryophyllene | 13.5 | 1428 | 1424 |
| α-*trans*-bergamotene | 0.6 | 1442 | 1432 |
| α-humulene | 5.3 | 1462 | 1454 |
| 9-*epi*-*E*-caryophyllene | 0.7 | 1470 | 1464 |
| γ-muurolene | 0.5 | 1485 | 1478 |
| α-amorphene | 0.2 | 1488 | 1482 |
| β-selinene | 1.5 | 1495 | 1492 |
| α-selinene | 1.7 | 1504 | 1501 |
| *Z*-γ-bisabolene | 1.4 | 1516 | 1511 |
| γ-cadinene | 0.4 | 1523 | 1512 |
| selina-4(15),7(11)-diene | 1.3 | 1545 | 1540 |
| selina-3,7(11)-diene | 2.5 | 1552 | 1546 |
| Germacrene-B | 0.3 | 1569 | 1557 |
| caryophyllene oxide | 2.2 | 1599 | 1587 |
| humulene epoxide | 0.7 | 1626 | 1613 |
| α-bisabolol | 0.5 | 1697 | 1688 |
| tetracosane | 6.0 | 2406 | 2400 |
| heptacosane | 23.9 | 2690 | 2700 |
| 12 unknown compounds | 4.8 | | |

[a]calculated retention index (RI). [b]retention index reported in literature (RIL) or in commercial databases

**Table S2.** GC-MS analysis of *Futura 75* essential oil.

| Compound | Area % | RI[a] | RIL[b] |
|---|---|---|---|
| α-pinene | 14.9 | 934 | 933 |
| camphene | 0.3 | 949 | 953 |
| β-pinene | 3.8 | 977 | 978 |
| myrcene | 11.8 | 994 | 991 |
| α-phellandrene | 0.2 | 1006 | 1007 |
| $\Delta^3$-carene | 0.5 | 1011 | 1009 |
| α-terpinene | 0.2 | 1018 | 1018 |
| *para*-cymene | 0.1 | 1028 | 1025 |
| limonene | 1.8 | 1031 | 1030 |
| eucalyptol | 0.2 | 1041 | 1032 |
| *E*-β-ocimene | 2.9 | 1053 | 1046 |
| γ-terpinene | 0.2 | 1062 | 1058 |
| terpinolene | 5.1 | 1091 | 1086 |
| *Z*-caryophyllene | 0.5 | 1414 | 1413 |
| α-cis-bergamotene | 0.3 | 1422 | 1416 |
| *E*-caryophyllene | 19.3 | 1428 | 1424 |
| α-*trans*-bergamotene | 1.9 | 1442 | 1432 |
| α-humulene | 8.3 | 1462 | 1454 |
| 9-*epi*-caryophyllene | 1.1 | 1470 | 1464 |
| β-selinene | 1.7 | 1495 | 1492 |
| α-selinene | 1.3 | 1504 | 1501 |
| selina-4(15),7(11)-diene | 0.9 | 1545 | 1540 |
| selina-3,7(11)-diene | 1.5 | 1552 | 1546 |
| caryophyllene oxide | 4.3 | 1599 | 1587 |
| humulene epoxide | 1.1 | 1626 | 1613 |
| *allo*-aromadendrene epoxide | 0.4 | 1650 | 1644 |
| tetracosane | 8.8 | 2407 | 2400 |
| 13 unknown compounds | 6.6 | | |

[a]calculated retention index (RI). [b]retention index reported in literature (RIL) or in commercial databases

**Table S3.** GC-MS analysis of *Carmagnola selezionata* essential oil.

| **Compound** | **Area %** | **RI[a]** | **RIL[b]** |
|---|---|---|---|
| α-pinene | 12.6 | 934 | 933 |
| camphene | 0.3 | 949 | 953 |
| β-pinene | 4.1 | 977 | 978 |
| myrcene | 26.4 | 995 | 991 |
| α-phellandrene | 0.3 | 1006 | 1007 |
| $\Delta^3$-carene | 0.3 | 1012 | 1009 |
| α-terpinene | 0.3 | 1019 | 1018 |
| *para*-cymene | 0.2 | 1028 | 1025 |
| limonene | 4.7 | 1031 | 1030 |
| eucalyptol | 0.5 | 1041 | 1032 |
| *Z*-β-ocimene | 0.4 | 1043 | 1035 |
| *E*-β-ocimene | 2.5 | 1053 | 1046 |
| γ-terpinene | 0.3 | 1062 | 1058 |
| terpinolene | 7.0 | 1091 | 1086 |
| linalyl anthranilate | 0.3 | 1110 | 1104 |
| fenchyl-alcohol | 0.2 | 1123 | 1123 |
| *Z*-caryophyllene | 0.4 | 1414 | 1413 |
| *E*-caryophyllene | 19.1 | 1428 | 1424 |
| α-*trans*-bergamotene | 0.2 | 1442 | 1432 |
| α-humulene | 7.2 | 1462 | 1454 |
| 9-*epi*-*E*-caryophyllene | 0.6 | 1470 | 1464 |
| β-selinene | 1.3 | 1495 | 1492 |
| α-selinene | 1.1 | 1504 | 1501 |
| *Z*-γ-bisabolene | 1.1 | 1514 | 1511 |
| selina-4(15),7(11)-diene | 0.3 | 1545 | 1540 |
| selina-3,7(11)-diene | 0.5 | 1552 | 1546 |
| caryophyllene oxide | 3.2 | 1599 | 1587 |
| humulene epoxide | 1.0 | 1626 | 1613 |
| 10 unknown compounds | 3.6 | | |

[a]calculated retention index (RI). [b]retention index reported in literature (RIL) or in commercial databases
